# Analytical Applications of Reactions of Iron(III) and Hexacyanoferrate(III) with 2,10-Disubstituted Phenothiazines

**DOI:** 10.1155/2009/302696

**Published:** 2009-02-02

**Authors:** Helena Puzanowska-Tarasiewicz, Joanna Karpińska, Ludmiła Kuźmicka

**Affiliations:** Institute of Chemistry, University of Białystok, Hurtowa 1, 15-399 Bialystok, Poland

## Abstract

The presented review is devoted to analytical applications of reactions of Fe(III) and K_3_[Fe(CN)_6_] with 2,10-disubstituted phenothiazines (PT). It was found that iron(III) and hexacyanoferrate(III) ions in acidic media easily oxidized PT with the formation of colored oxidation products. This property has been exploited for spectrophotometric determination of iron(III) ions and phenothiazines. Some flow-injection procedures of the determination of PT based on the oxidation reaction by means of the above-mentioned oxidants have been proposed. In the presented review, the application of 2,10-disubstituted phenothiazines as indicators in complexometric titration of iron(III) as well as procedures of PT determination based on generation of ternary compound in the system Fe(III)-SCN^−^- PT was also described.

## 1. Introduction

Phenothiazines constitute one of the largest chemical classes of organic compounds in official compendia. Over 4 thousands compounds have been synthesized and about 100 have been used in clinical practice [1]. 2,10-Disubstituted phenothiazines are very important drugs which are widely used in psychiatric treatment as tranquillizers. Invention and introduction of phenothiazine derivatives into treatment of mental disease has changed the modern psychiatry. This fact has improved the life style of patients and allowed quick development of ambulatory system of treatment for such sickness. The common use of phenothiazines has generated the need for fast and reliable methods for quality control of phenothiazine pharmaceuticals and monitoring them in clinical samples. Over fifty years of the medical use of phenothiazines have resulted in countless number of analytical procedures devoted to resolve this problem [2, 3].

Phenothiazine derivatives are interesting from analytical point of view due to their characteristic structure—the presence of chemically active sulfur and nitrogen atoms in positions 5 and 10 and substituents in position 2 and alkylamine side chain at N_10_ atom. Phenothiazine and its derivatives are characterized by low ionization potentials [1, 4]. They are easily oxidized by different chemical, electrochemical, photochemical, and enzymatic agents with the formation of colored oxidation product—intermediate cation radical [1]. Colors of formed intermediate depend on a presence and a structure of substituents in positions 2 and 10 (Table 1).

The run of reactions with the oxidants (e.g., Fe(III), [Fe(CN)_6_]^3−^, Cr_2_O_7_^2−^, IO_3_^−^, IO_4_^−^, BrO_3_^−^, H_2_O_2_, chloramine T) has been studied and employed for determination of used phenothiazine or oxidant. The stability of oxidation products depends on acidity, concentration of oxidizing agents, time, temperature, and the presence of some salts [5]. Recently the redox properties of 2,10-disubstituted phenothiazines radicals have been studied by Madej and Wardman [6]. They have established the reduction potentials of phenothiazine radicals and equilibrium constants using a pulse radiolysis and a cyclic voltammetry. The further oxidation leads to a generation of colorless sulphoxide. A lot of published works have been based on the oxidation behavior of 2,10-disubstituted phenothiazines [1, 7, 8]. The oxidation involves a series of one-electron steps providing free radicals and cations [1]. The distribution of *π*-electrons in the 2,10-disubstituted phenothiazines, according to theoretical considerations, may lead to the formation of some resonance forms of free cation radical [9].

Phenothiazines have exhibited complexing properties due to the presence of the condensed three-ring aromatic system and amine nitrogen atom in a side chain in position 10. They have reacted with some metal ions or thiocyanate complexes of metals forming colored, hard soluble in water but easy soluble in organic solvents compounds [1, 10]. Some organic substances (e.g., picric, flavianic acid, alizarin S, brilliant blue, pyrocatechol violet) have formed with 2,10-disubstituted phenothiazines colored ion-association compounds sparingly soluble in water, but quantitatively extracted into organic phase [10, 11]. The conducted spectroscopic studies have confirmed ion-association nature of these conjunctions [10, 11]. Phenothiazines also have created charge-transfer complexes with nitroso-R-salt [12] and chloranilic acid [13].

In our earlier works, it has been found that 2,10-disubstituted phenothiazines are useful as redox indicators [8, 19] and spectrophotometric reagents [10, 11, 20]. Some elements have a catalytic influence on a run of oxidation of phenothiazines [21, 22]. The catalytic effect of presence of iodide, nitrite, vanadium, and iron ions on the reactions of phenothiazines with KBrO_3_, H_2_O_2_ has been described by Mohamed [23] in his dissertation.

After careful analysis of articles concerned with phenothiazines determination, it could be stated that iron ions and its anionic complexes are reagents most often used for this purpose. The mild oxidation potential of Fe(III)/Fe(II) couple and stability of its complexes make them very convenient reagents for phenothiazine derivatives assay. The oxidation reaction conducted in the system Fe(III)-NO_3_^−^-CIO_4_^−^ (Forrest's reagent) is still used for quick examination of presence of phenothiazines in studied sample [24]. Taking the above-mentioned facts into account, we have decided to get together the most important information focused on analytical applications of the reactions of 2,10-disubstituted phenothiazines with Fe(III) and [Fe(CN)_6_]^3−^ ions.

## 2. Reactions of Phenothiazines with Iron(III) and Hexacyanoferrate(III) Ions

As mentioned above, the most important property of phenothiazines is their susceptibility to oxidation by many oxidizing agents, for example, Fe(III), [Fe(CN)_6_]^3−^ with the formation of colored oxidation products (free radicals) in acidic media [8, 21]. The stability of the oxidation products depends on the nature of the substituents at positions 2 and 10 (Table 1) [25]. The stability of 57 radicals has been studied by Levy et al. [26] using ESR method and the Hammett metasubstituent constant. The kinetics [27] of oxidation reactions of eight phenothiazines with [Fe(H_2_O)]^3+^ and [Fe(CN)_6_]^3−^ and an influence of micellar system [28] on the run of studied processes have been investigated by Pelizzetti and Mentasti [27] and Pelizzetti et al. [28].

Basavaiah and Swamy [29] have studied oxidation reaction of five phenothiazine derivatives with hexacyanoferrate(III). The reduced hexacyanoferrate(II) reacted further with ferrin forming ferroin. The measurements of absorbance of final product allowed to determine studied compounds in the concentration range 1–12 *μ*g/mL with the molar absorption coefficient ranged from 2.08 × 10^4^ for chlorpromazine to 3.49 × 10^4^ for prochlorpromazine.

**Figure 1:**
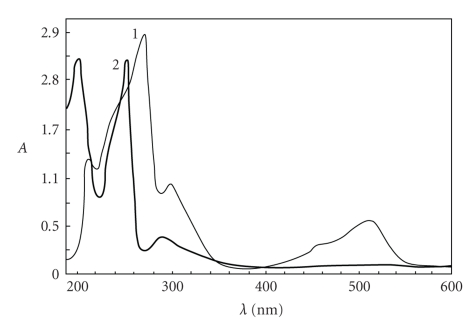
UV-VIS spectra of 1-colored product of reaction PM-Fe(III) (C_PM_ = 3·10^−4^ M, C_Fe(III)_ = 3·10^−4^ M; 2-nonoxidized form of PM (C_PM_ = 4·10^−4^ M).

In our previous works [14, 15], we have described the optimal conditions for the formation of colored products of 2,10-disubstituted phenothiazines with FeCl_3_ and K_3_[Fe(CN)_6_] in acidic media. The absorption spectra of these products in aqueous solutions have been recorded. The spectra of the nonoxidized and colored oxidation product, for example, promazine hydrochloride (PM) are given in Figure 1. We did not obtain the spectrum of sulphoxides using FeCl_3_ [14] and K_3_[Fe(CN)_6_] [15] as oxidants.

**Scheme 1:**
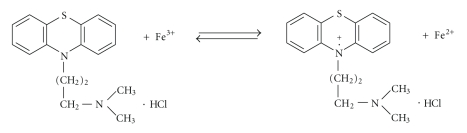
The oxidation of PM by means of FeCl_3_.

The investigations in the UV-region have testified that the reaction of PT with FeCl_3_ or K_3_[Fe(CN)_6_] proceeds only to the first steep, for example, promazine (Scheme 1).

**Figure 2:**
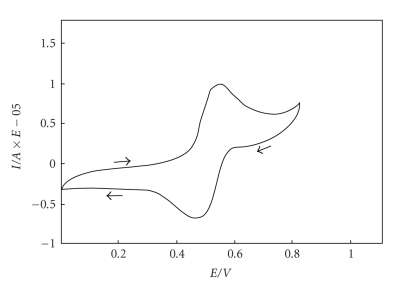
Cyclic voltammogramm of promazine (PM) at Pt electrode C_PM_ = 5·10^−5^ M, in a mixture of 5·10^−1^ M K_2_SO_4_ and 5·10^−1^ M KHSO_4_ (pH = 1.3), scan rate 50 mV/s; in potential window 0.0–1.05 V.

The presence of iron(II) after the oxidation of phenothiazines studied has been confirmed by means of 2,2′-bipirydyl [15, 17]. A reversible nature of oxidation process of phenothiazines has been reentered by the electrochemical method—cyclic voltammetry [14]. Figure 2 shows a cyclic voltammogramm for promazine. As can be seen, promazine exhibits an oxidative peak at 0.55 V. Acorresponding reductive peak (0.47 V) appears when the polarization of electrode is reversed. This suggests that the reaction can be regarded as reversible (Scheme 1).

From already used various oxidants, iron(III) ion has been chosen as a mild oxidation agent for spectrophotometric determination of 2,10-disubstituted phenothiazines. The formal redox potential of the Fe(III)-Fe(II) couple (*E*° = 0.77 V, v SME) does not allow further oxidation of the colored free radicals to the uncolored sulphoxides [9]. The formed oxidation products are stable. These properties have been exploited in chemical analysis [1, 8, 19, 20]. The oxidation properties of iron(III)-phenothiazines system have been employed for the spectrophotometric determination of iron(III) ions [16] and diethazine [18].

## 3. Application of the Reactions in FIA Systems

Some flow injection procedures have been described for the determination of PT. They have been based on the oxidation reaction of 2,10-disubstituted phenothiazines with iron(III) or hexacyanoferrate(III) (Table 2).

**Figure 3:**
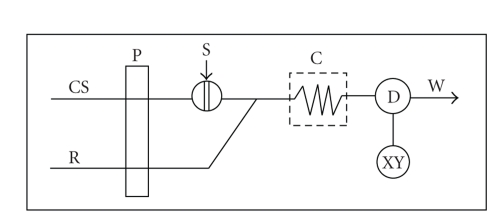
Schematic diagram of the FIA manifold used for the assay of promazine; R: iron(III) in hydrochloric acid solution; CS: water; P: peristaltic pump; S: samples injector; C: reactor; D: spectrophotometer adjusted to the corresponding wavelength of the oxidized form of phenothiazines; X: recorder; W: waste.

Phenothiazines solutions have been injected into a stream of distilled water, which has been merged with the stream of iron(III) chloride in hydrochloric acid [30] or with the stream of iron(III) perchlorate in perchlorate acid medium [31]. One of the used FIA manifolds is presented in Figure 3.

Another method for determination of promazine [32] has been proposed by Kojło et al. This assay has been based on promazine oxidation by K_3_[Fe(CN)_6_] previously retained on anion exchange column. The oxidation has been carried out at room temperature in aqueous acidic medium.

The flow injection (FIA) methods are preferable to other conventional methods because they are fast (from 50 to 200 samples studied per h) and precise (RSD values ranging from 0.6 to 2.5%). Additional advantage of flow methods is a possibility of combination with preconcentration of assayed phenothiazine on sorption column mounted on line [33]. Another applied approach is the use a detection cell filled with suspension of an appropriate sorbent (so-called a solid spectrophotometry mode) [34].

## 4. Application of Phenothiazines As Visual Indicators in Complexometric Titration

2,10-Disubstituted phenothiazines are useful redox indicators. The radical cations, which are stable enough under acidic conditions, exhibit quite intense color [1, 4, 6]. This property allows employing phenothiazines as redox indicators in many redoxometric determinations. The values of reduction potentials of some PT established by Madej and Wardman [6] and Gowda and Ahmed [35] are given in Table 3.

Phenothiazines have been used as indicators for complexometric determination of iron(III) with dissodium versenate [36]. They form with Fe(III) ions colored oxidation products (red, orange, or blue). The addition of dissodium versenate to the titrated solution containing iron(III) solution and phenothiazines as indicator has caused a change of the test solution in end point of titration as shown in Table 4.

The usefulness of PT (chlorpromazine, promazine, perphenazine, methopromazine) as redox indicators in chromatometric determination of K_4_[Fe(CN)_6_] has been described by Puzanowska-Tarasiewicz et al. [38].

Phenothiazines indicators are superior to conventional indicators (e.g., ferroin, variamin blue). They give sharper end point and act over a wider range of acidity than other conventional indicators.

## 5. Application of Complexation Reactions in Assay of Phenothiazines

As it was mentioned in introduction section, phenothiazines show the ability to create stable compounds in reaction with anionic complexes of metal ions, some organic anions or with *π*-electron acceptors.

According to Ozutsumi et al. [39], the formation of the thiocyanate-iron(III) complexes in aqueous solution and the development of the red color are related to [Fe(SCN)]^2+^, [Fe(SCN)_2_]^+^, [Fe(SCN)_3_], [Fe(SCN)_4_]^−^, [Fe(SCN)_5_]^2−^, [Fe(SCN)_6_]^3−^. Tarasiewicz [37] has found that one of these complexes reacts with 2,10-disubstituted phenothiazines forming red-brown compounds. The optimal conditions for the formation of the compounds have been established and the composition determined. The absorption spectra have been recorded in UV-VIS and IR regions [37]. On the basis of obtained data, the following reaction course in PT-Fe(III)-SCN^−^ system has been suggested:

(1) $$\begin{array}{cc} \text{Fe(III)}+m\;{\text{SCN}}^{-} & \to {\left[\text{Fe}{\left(\text{SCN}\right)}_{m}\right]}^{n-},\\ \text{PT}+{\text{H}}^{+} & \to {\left(\text{PT}•\text{H}\right)}^{+},\\ {\left(\text{PT}•\text{H}\right)}^{+}+{\left[\text{Fe}{\left(\text{SCN}\right)}_{\text{m}}\right]}^{n-} & \to {\left(\text{PT}•\text{H}\right)}_{n}[\text{Fe}{\left(\text{SCN}\right)}_{m}], \end{array}$$

where*n* = 2, *m* = 5.

The spectral properties of these compounds suggest that the formation of color compound occurred due to interaction between opposite charged ions (large phenothiazines cation and an anionic thiocyanate complex of a metal). The obtained color precipitate is quantitatively extracted with chloroform or dissolved in acetone with formation colored and stable solution. This property has been the basis of sensitive extractive-spectrophotometric or spectrophotometric method of determination of phenothiazines [37] (Table 5).

Chlorpromazine and some phenothiazines react with ferro- and ferricyanate ions [40] and nitroso-ferricyanate [41] to form ion association-compounds sparingly soluble in water. The composition of these compounds has been established and physicochemical properties have been investigated.

Valero [42] has stated, using UV-VIS spectrophotometry, that iron(III) forms ternary complexes with pyrocatechol violet (PCV) and chlorpromazine (CPZ) which compositions have been established as: Fe:PCV:CPZ = 1:2:3 and Fe:PCV:CPZ = 1:3:4. The last complex can be used for spectrophotometric determination up to 1.6 ppm of iron(III).

## 6. Other Applications

Some ions of *d*-electron elements exhibit a catalytic effect on the oxidation of 2,10-disubstituted phenothiazines [21]. Fukasawa et al. [22] have described a spectrophotometric determination of trace amounts of iron by its catalytic effect on the thioridazine-H_2_O_2_ reaction. It was found that others *d*-electron ions of metals have the catalytic effect on phenothiazine reactions with H_2_O_2_ [21].

**Scheme 2:**
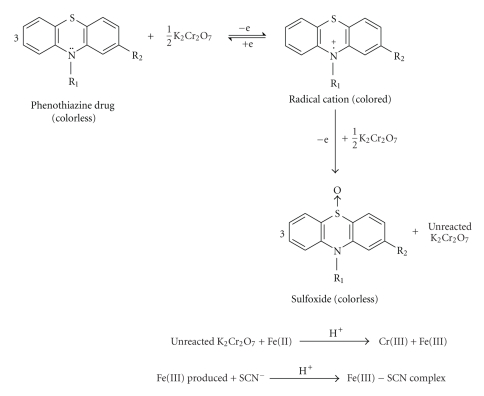
Reaction scheme showing the formation of iron(III)-thiocyanate complex and correlation of the latter's concentration with phenothiazine drugs concentration.

It is known that stability of color cation radical depends mainly on oxidation agent used. In the case of strong oxidant, the color of radical disappears quickly due to the second step of reaction which leads to the formation of a colorless sulphoxide. This effect resulted in decrease of sensitivity of assay and reproducibility. In purpose to improve these analytical properties indirect methods of phenothiazines determination have been proposed. One of them has been described by Basavaiah and Swamy [43]. They have applied potassium dichromate and iron-thiocyanate for spectrophotometric investigations of phenothiazines (chlorpromazine, promethazine, triflupromazine, trifluoperazine, fluphenazine, prochlorperazine). They have used a combination of dichromate and iron(III)-thiocyanate system for the determination of phenothiazines (Scheme 2).

Potassium dichromate as a strong oxidizing agent (couple Cr_2_O_7_^2−^/Cr^3+^ *E* ^0^ = 1.33 V, *versus* standard hydrogen electrode) oxidizes 2,10-disubstituted phenothiazines via colored radical cation to a colorless sulphoxide [43]. The excess of used oxidant has been further reduced by iron(II) ions. Next, the thiocyanate ions have been used for quantification of produced iron(III) ions. It has been stated that the absorbance of iron(III) thiocyanate solution is proportional to amount of the determined phenothiazines. The sensitivity of the proposed method has been the best at the molar ratio K_2_Cr_2_O_7_:PT equal to 1:6 at room temperature [43].

## 7. Conclusions

Based on information gathered in the presented review, it can be concluded that iron(III) ion and its anionic complexes are valuable reagents useful in an analysis of phenothiazines (PT). The mild oxidation potential of iron(III) and K_3_[Fe(CN)_6_] allows quantification of phenothiazines in batch and flow systems. The proposed methods are characterized by simplicity, sensitivity, and good precision. The determination of PT by flow injection (FIA) methods is preferable to other conventional methods because they are fast (from 50 to 200 samples studied per hour) and precise (RSD values ranging from 0.6 to 2.5%).

The ability to crate ion-pair compounds can be employed for selective and sensitive determination of iron ions(III) and phenothiazines as well.

## Figures and Tables

**Table 1:** The structures of 2,10-disubstituted phenothiazines studied with Fe(III) and K_3_[Fe(CN)_6_] and colors of their cation radicals.

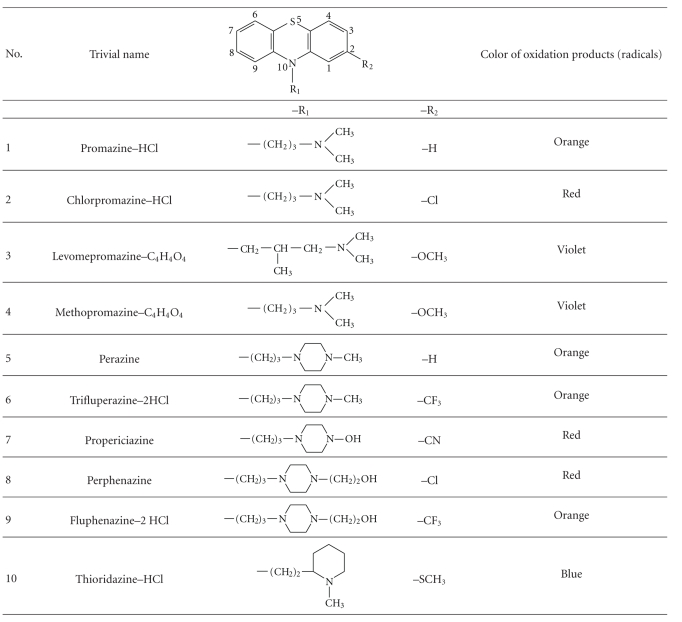

**Table 2:** Determination of some PF with Fe(III) and K_3_[Fe(CN)_6_] using flow injection methods.

Oxidant	Compound	Determination range (*μ*g mL^−1^)	Pharmaceutical formulation	Ref.
FeCl_3_	Promazine	10–130		
	Thioridazine	10–130	Promazin (injection)	
				[14]
Fe(CIO_4_)_3_	Promazine	6–128		
	Chlorpromazine	6–124	Largactil (tablets)	
	Levomepromazine	7–133		
	Promethazine	6–117		[15]
	Fluphenazine	5–312	Phenergan (tablets)	
	Thioridazine	11–230	Majeptil (tablets)	
	Thioproperazine	12–248		[15]
	Trifluoperazine	12–230		
	Promethazine	0,5–8,0	Syrup, cream and tablets	[16]
	Trifluoperazine	0,5–10		
K_3_[Fe(CN)_6_]	Promazine	2,5–25	Promazine (injection)	[17]
	Chlorpromazine	1–2	Urine	
	Fluphenazine			
	Thioridazine			[18]
	Promethazine			
	Methotrimeprazine			

**Table 3:** Reduction potentials of PT.

Phenothiazines	mV	Phenothiazines	mV
Chlorpromazine*	860	Propericiazine*	966
Promethazine*	925	Trifluoperazine	880
Diethazine	845	Prochlorperazine	799
Thioridazine*	789	Butaperazine	865

*Determined by Madej and Wardman at pH ∼ 5–7 [6]; others values established by Kojło et al. in 0,5 M H_2_SO_4_ [32].

**Table 4:** 

Colorless → red	Colorless → orange	Colorless → blue
*Chlorpromazine*	*Propericiazine*	*Thioridazine*
*Diethazine*	*Trifluoperazine*	
*Promethazine*	*Prochlorperazine*	
	*Butaperazine*	

**Table 5:** Extractive-spectrophotometric and spectrophotometric methods of determination of some of 2,10-disubstituted phenothiazines.

Organic phase	Phenothiazines	Determination range(*μ*g mL^−1^)	Ref.
Chloroform	Chlorpromazine	120–300	
	Levomepromazine	140–400	[37]
	Promethazine	160–550	

Acetone	Chlorpromazine	20–150	
	Levomepromazine	20–300	[37]
	Promethazine	60–400	
